# Anesthesia-induced atelectasis assessed by lung sonography

**DOI:** 10.1186/2036-7902-6-S1-A13

**Published:** 2014-01-31

**Authors:** CM Acosta, G Tusman, D Jacovitti, G Maidana, A Belaunzarán, S Cereceda, E Rae, A Molina, S Gonorazky

**Affiliations:** 1 Department of Anesthesia, Hospital Privado de Comunidad, Mar del Plata, Buenos Aires, Argentina; 2 Department of Radiology, Hospital Privado de Comunidad, Mar del Plata, Buenos Aires, Argentina; 3 Department of Clinical Research, Hospital Privado de Comunidad, Mar del Plata, Buenos Aires, Argentina

## Background

Atelectasis and poorly ventilated lung areas are negative consequences of general anesthesia observed in adult as well as in children. The reported incidence of this anesthesia-induced atelectasis in children is high and comes from 83 % to almost 100 %. The diagnosis of this entity is difficult to do at the bedside; they are commonly small and mostly invisible to standard chest X-ray images. More complex diagnosing techniques like CT scan or magnetic resonance imaging (MRI) can easily diagnose atelectasis although they are strategically impractical. Ultrasound is a bedside, rapid, noninvasive and radiation-free technique. Lung ultrasound (LUS) has an important role for diagnosing pulmonary diseases in children although it role for detecting anesthesia-induced atelectasis has not been determined yet.

## Objective

The aim of this study was to test the accuracy of LUS to detect anesthesia-induced atelectasis in children undergoing magnetic resonance imaging (MRI) studies.

## Patients and methods

**Figure 1:**
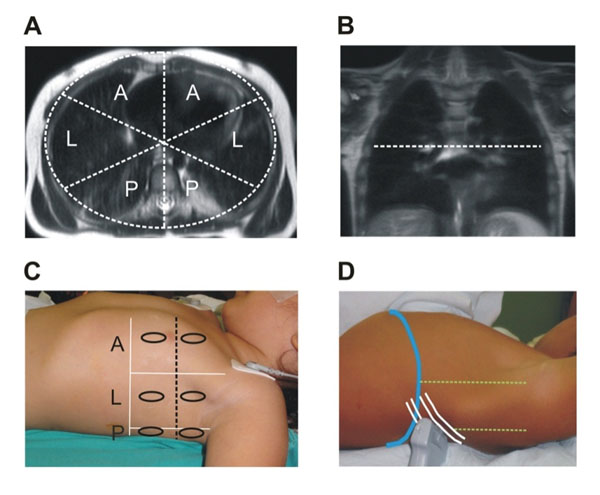
Chest segmentation of MRI-LUS image. A) The axial MRI cuts were segmented in anterior (A), lateral (L) and posterior (P) regions in both lungs. B) The coronal MRI view was used to divide the lungs in superior and inferior lungs regions. C) The LUS assessment followed similar chest regions than MRI images. The dotted black axial line separated the lungs in superior and inferior portions. Ovals depicted where the probe is placed in the classical LUS approach: mid-clavicular, lateral and posterior axillary lines. D) The posterior regions were also assessed by the inferior postero-basal LUS view.

A total of fifteen children (6 females and 9 males), American Society of Anesthesiologists physical status I criteria, (ASA I), aged 1-7 years old were studied. LUS was performed with a linear probe of 6-12 MHz in anesthetized children undergoing MRI studies after the reference lung MRI images were taken. The presence and distribution of atelectasis was described segmenting the chest in 12 similar anatomical regions for both methods (Figure 1). The analysis of images was performed by 4 independent radiologists, 2 for LUS and 2 for MRI. The level of agreement for the diagnosis of atelectasis among observers was analyzed using the κ reliability test.

**Table 1:** Prevalence of LUS signs related to anesthesia induced atelectasis in children

LUS sign	Observer 1 *Total observations = 55*		Observer 2 *Total observations = 50*	
	
	*Number of observations*	*Percentage of total - IC*	*Number of observations*	*Percentage of total-IC*
Irregular pleural line	52	95 % (84-98)	49	98 % (89-99)

Absence of A lines	49	89 % (75-94)	44	88 % (73-94)

Presence of B lines	50	91 % (77-95)	45	90 % (78-96)

Air bronchograms	38	69 % (53-79)	35	70 % (55-82)

Absence of lung sliding	22	40 % (27-54)	22	44 % (29-58)

Presence of the pulse sign	22	40 % (27-54)	22	44 % (29-58)

**Figure 2:**
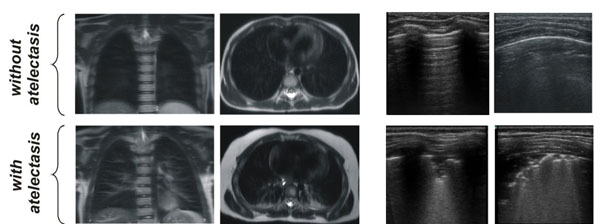
Example of patients with and without atelectasis. **Left:** MRI coronal and axial images used as reference method to detect anesthesia-induced atelectasis. **Right:** Atelectasis induced by anesthesia is determined by LUS as irregular pleural line and subpleural consolidation with air bronchograms and trailing B lines.

## Results

Fifteen patients were successfully studied. Pairs of MRI and LUS images that belong to 180 lung regions (12 regions per patient in a total of 15 patients) were included in the analysis. Fourteen patients (93%) developed atelectasis in 39 of their 168 lung regions observed with MRI images. Most of them were crescent-like or sub-segmental atelectasis born from the sub-pleural areas. LUS detected atelectasis in the same 14 patients diagnosed by MRI. Anesthesia-induced atelectasis prevail in the dependent areas of lungs. The irregular pleural line was the most common LUS sign of atelectasis observed by both radiologists. Contrarily, the lack of lung sliding and the presence of the pulse sign were the less observed LUS signs. LUS showed 90% of sensitivity (95% confidence interval 76-97%), 89% of specificity (95% confidence interval 83-94%) and 89% of accuracy (95% confidence interval 84-93%) for the diagnosis of atelectasis taking MRI as reference. We found a very good agreement between 2 radiologists for diagnosing atelectasis by RMI (κ 0.87, 95% confidence interval 0.72-1; p < 0.0001) and 2 radiologists for detecting atelectasis by LUS (κ 0.93, 95% confidence interval 0.79-1; p < 0.0001). MRI and LUS exhibited good agreement when data from the four radiologists were pooled and examined together (κ 0.75, 95% confidence interval 0.69-0.81; p < 0.0001).

## Conclusion

Lung sonography is an accurate, safe and simple method for diagnosing anesthesia-induced atelectasis in children at the bedside. This tool would help anesthesiologists to treat this entity and to adjust the ventilatory setting during surgery.
